# Effect of Graphene Oxide on the Reaction Kinetics of Methyl Methacrylate In Situ Radical Polymerization via the Bulk or Solution Technique

**DOI:** 10.3390/polym9090432

**Published:** 2017-09-08

**Authors:** Ioannis S. Tsagkalias, Triantafyllos K. Manios, Dimitris S. Achilias

**Affiliations:** Laboratory of Polymer Chemistry and Technology, Department of Chemistry, Aristotle University of Thessaloniki, 541 24 Thessaloniki, Greece; itsagkal@chem.auth.gr (I.S.T.); trfgr1@gmail.com (T.K.M.)

**Keywords:** PMMA, graphene oxide, polymerization kinetics, bulk, solution

## Abstract

The synthesis of nanocomposite materials based on poly(methyl methacrylate) and graphene oxide (GO) is presented using the in situ polymerization technique, starting from methyl methacrylate, graphite oxide, and an initiator, and carried out either with (solution) or without (bulk) in the presence of a suitable solvent. Reaction kinetics was followed gravimetrically and the appropriate characterization of the products took place using several experimental techniques. X-ray diffraction (XRD) data showed that graphite oxide had been transformed to graphene oxide during polymerization, whereas FTIR spectra revealed no significant interactions between the polymer matrix and GO. It appears that during polymerization, the initiator efficiency was reduced by the presence of GO, resulting in a reduction of the reaction rate and a slight increase in the average molecular weight of the polymer formed, measured by gel permeation chromatography (GPC), along with an increase in the glass transition temperature obtained from differential scanning calorimetry (DSC). The presence of the solvent results in the suppression of the gel-effect in the reaction rate curves, the synthesis of polymers with lower average molecular weights and polydispersities of the Molecular Weight Distribution, and lower glass transition temperatures. Finally, from thermogravimetric analysis (TG), it was verified that the presence of GO slightly enhances the thermal stability of the nano-hybrids formed.

## 1. Introduction

Graphene is a single-atomic, two-dimensional layer of sp^2^ hybridized carbon atoms arranged in a honeycomb lattice. Because of its unique mechanical, electrical, thermal, and optical properties, it has recently attracted the research interest of the scientific community [1,2]. These properties make graphene one of the most popular candidates for the development of functional and structural graphene-reinforced polymer composites. Graphene can be obtained from the exfoliation of graphite sheets. However, it is easier to obtain graphene oxide (GO) sheets through the exfoliation of graphite oxide. The latter can be produced by the oxidation of graphite and consists of many oxygen-containing groups, such as carboxyl, epoxy, and hydroxyl groups in the basal planes and edges [2,3]. Thus, graphite oxide exhibits an increased interlayer spacing from the original 3.4 Å of graphite to 6.0–10 Å [3]. Such functional groups aim to produce graphite oxide hydrophilic and to weaken the van der Waals forces between layers. Thus, graphite oxides can be readily dispersed in aqueous media to form colloidal suspensions. This facilitates the exfoliation of layered graphite oxide into GO sheets via sonication or stirring [4].

Various techniques have been developed for the synthesis of polymers based on nano-composites, including solution mixing, melt blending, and in-situ polymerization [5,6,7]. The latter technique usually ensures the good dispersion of the nano-additive in the polymer matrix and has improved the final product properties. The in situ polymerization in the presence of several nano-additives was also the basis of an extensive experimental study conducted by our group [8,9,10,11,12].

Although nanocomposites of GO with several polymers have been the subject of studies in the past [1,7], their incorporation in methyl methacrylate (MMA) polymerization under bulk or solution conditions has not yet appeared in scientific papers and journals [5,6]. Therefore, the challenge in this work is to experimentally investigate the kinetics of the in-situ polymerization of MMA in the presence of a material having unique properties, such as the graphene oxide (GO) nano-additive. GO was formed during the reaction by the exfoliation of graphite oxide obtained from the oxidation of graphite. One of the major problems in such polymerizations is the formation of stable dispersion throughout the reaction. For this reason, solvents are usually chosen. Dimethylformamide (DMF) is an organic solvent which dissolves both the monomer MMA and the polymer PMMA and is also hydrophilic and polar with a rather high boiling point (153 °C). It has been proved to be a good solvent for the dispersion of GO [13]. Therefore, in this research, besides carrying out the polymerization in bulk, the possibility of using DMF as a reaction solvent was examined. Thus, for the first time, the effect of GO on the in situ solution polymerization of MMA was explored.

In the past, we have prepared nanocomposite materials of PMMA with several nano-clays in our laboratory and studied the influence of the nano-filler on the reaction kinetics [14,15,16]. As a continuation of this work, nanocomposite materials of PMMA with graphene oxide are produced in this study using an in-situ polymerization technique carried out with (solution) or without (bulk) in the presence of a solvent. The effect of GO on the reaction kinetics is investigated gravimetrically by measuring the variation of conversion with time, as well as the molecular weight distribution of the polymer formed. The properties of the PMMA/GO nanohybrids were measured via a variety of techniques such as X-ray diffraction, FTIR spectrometry, thermogravimetric analysis (TGA), gel permeation chromatography (GPC), and differential scanning calorimetry (DSC).

## 2. Materials and Methods

### 2.1. Materials

Methyl methacrylate, used as the monomer, was purchased from Alfa Aesar (Haverhill, MA, USA purity ≥ 99%). The inhibitor, hydroquinone, was removed by passing the monomer before any use, thrice, through disposable inhibitor-remover packed columns (Aldrich, Hamburg, Germany). Benzoyl peroxide (BPO) was used as a free radical initiator and was provided by Alfa Aesar (purity > 97%). The initiator was purified by fractional recrystallization twice from methanol (Chem Lab, Zedelgem, Belgium). Dimethylformamide (DMF) was used as a solvent for the solution polymerization and as a means of exfoliation of graphite oxide to graphene oxide, and was supplied from the J.T. Baker company (Radnor, PA, USA). Dichloromethane was used as the polymer solvent purchased from the company Chem Lab, while the methanol used for the precipitation of the polymer was also supplied by the same company. All other chemicals used were of analytical grade and were used as received without further purification.

### 2.2. Preparation of Graphite Oxide

Graphite oxide (GO) was prepared by oxidizing graphite powder which was purchased from Sigma-Aldrich (St. Louis, MO, USA), in accordance with the Hummers method. Accordingly, 10 g of commercial graphite powder was dispersed in sulfuric acid (230 mL) at 0 °C. Subsequently, 30 g of potassium permanganate (KMnO_4_) was slowly added to the suspension by controlling the addition rate and maintaining the temperature below 20 °C. Following this, the reaction mixture was cooled to 2 °C. Then, the mixture was removed from the ice bath and stirred with a magnetic stirrer at room temperature for 30 min. Subsequently, 230 mL of deionised water was added, again controlling the addition rate, while the temperature was kept below 20 °C. Thereafter, the mixture was resuspended under mechanical agitation for 15 min, followed by the addition of 1.4 L deionized water and 100 mL of hydrogen peroxide solution (30 wt %). The mixture was allowed to stand for 24 h. The GO particles that settled at the bottom were separated from the excess liquid by decantation. The gelatinous texture material was placed in an osmotic membrane, to stop the formation of precipitate BaSO_4_, which appeared during the addition of BaCl_2_ aqueous solution. The material remained in the membrane for about eight days. Finally, the final product was obtained by the freeze-drying method.

### 2.3. Preparation of the Initial Monomer/GO Mixtures

Three different relative amounts of GO to monomer, i.e., 0.1, 0.5, and 1.0 wt % were prepared. Monomer with graphite oxide (for bulk polymerization), or monomer with the solvent (DMF) and graphite oxide (for solution polymerization), underwent ultrasonication for one hour to ensure a satisfactory colloidal dispersion of graphite oxide in the solution, while the exfoliation of graphite oxide to graphene oxide started. In the final suspension, the initiator BPO 0.03 M was added and the mixture was degassed by passing nitrogen through it, after which it was immediately used. During solution polymerization, two different relative amounts of monomer/solvent were employed, i.e., 80:20 and 50:50 *v*:*v*.

### 2.4. Synthesis of PMMA/GO Nanocomposites by the In-Situ Bulk or Solution Radical Polymerization

Bulk free-radical polymerization was carried out in small test-tubes at a constant temperature of 80 °C for a suitable time. According to this technique, 1 cm^3^ of the pre-weighed mixture of monomer with the initiator and each amount of GO were placed into a series of 10 small test-tubes. They were degassed with nitrogen, sealed, and placed into a pre-heated reaction temperature bath. Each test-tube was removed from the bath at pre-specified time intervals, a few drops of hydroquinone were added in order to stop the reaction, and it was immediately frozen. The product was isolated after dissolution in dichloromethane (CH_2_Cl_2_) and precipitation in methanol. A different procedure for the isolation of the product was followed in the last samples of each experiment. Since polymerization had already finished and the product was a hard solid, the test-tubes were broken and nanocomposites were obtained as such. In this manner, the filler was enclosed in the polymer matrix. Subsequently, all isolated materials were dried to a constant weight in a vacuum oven at room temperature, weighed, and the degree of conversion was estimated gravimetrically.

Neat polymer was also synthesized under the above conditions and used as the reference material.

Exactly the same procedure was repeated in the solution polymerization experiments but by using the monomer MMA dissolved in DMF in all experiments.

### 2.5. Measurements

*X-ray diffraction*. The crystalline structure of the prepared PMMA/GO materials was characterized using X-ray diffraction (XRD) in a Rigaku Miniflex II instrument (Tokyo, Japan) equipped with a CuKα generator (λ = 0.1540 nm). The XRD patterns were recorded at the range 2θ = 5–65° and a scan speed of 2°·min^−1^.

*Fourier-Transform Infra-Red (FTIR).* The chemical structure of neat PMMA and PMMA/GO nanocomposites was established by recording their IR spectra. The spectrophotometer used was Spectrum 1 (Perkin Elmer, Waltham, MA, USA) equipped with an attenuated total reflectance (ATR) device. Spectra were recorded over the range 4000 to 600 cm^−1^ at a resolution of 2 cm^−1^ and 32 scans were averaged to reduce noise. Thin films were used for the measurements prepared in a hot hydraulic press and the instrument’s software was employed to identify characteristic peaks.

*Differential Scanning Calorimetry (DSC)*. The glass transition temperature, *T*_g_, of the material prepared, was measured using the DSC-Diamond (Perkin-Elmer). Samples of approximately 5–6 mg were used and sealed in standard Perkin-Elmer sample pans. The temperature program followed included, initially heating to 180 °C at a rate of 10 °C·min^−1^ to ensure the complete polymerization of the residual monomer, cooling to 30 °C, and heating again to 130 °C at a rate of 20 °C·min^−1^. The glass transition temperature was estimated from the second heating recordings.

*Gel Permeation Chromatography (GPC).* In order to estimate the average molecular weights and the full molecular weight distribution (MWD) of neat PMMA and the PMMA/GO nanocomposites, GPC was used. The chromatograph used was from Polymer Laboratories (Church Stretton, UK), model PL-GPC 50 Plus, and included an isocratic pump, three PLgel 5 μ MIXED-C columns in series, and a differential refractive index (DRI) detector. The elution solvent was tetrahydrofurane (THF) at a constant flow rate of 1 mL·min^−1^, and the entire system was kept at a constant temperature of 30 °C. All samples were dissolved in THF at a concentration of 1 mg·mL^−1^, filtered, and 200 μL was used for the injection into the chromatograph. Calibration of GPC was carried out with standard poly(methyl methacrylate) samples having peak molecular weights ranging from 690 to 1,944,000 (from Polymer Laboratories).

*Thermogravimetric Analysis (TGA).* The thermal stability of the samples was evaluated by measuring their mass loss with increasing temperature using TGA. Measurements were performed on a Pyris 1 TGA (Perkin-Elmer) thermal analyzer. Samples of approximately 8–10 mg were used and the measurements included heating from ambient temperature to 600 °C at a heating rate of 20 °C·min^−1^ under inert atmosphere (nitrogen flow).

## 3. Results

### 3.1. Characterization of the PMMA/GO Nanocomposites

**Figure 1:**
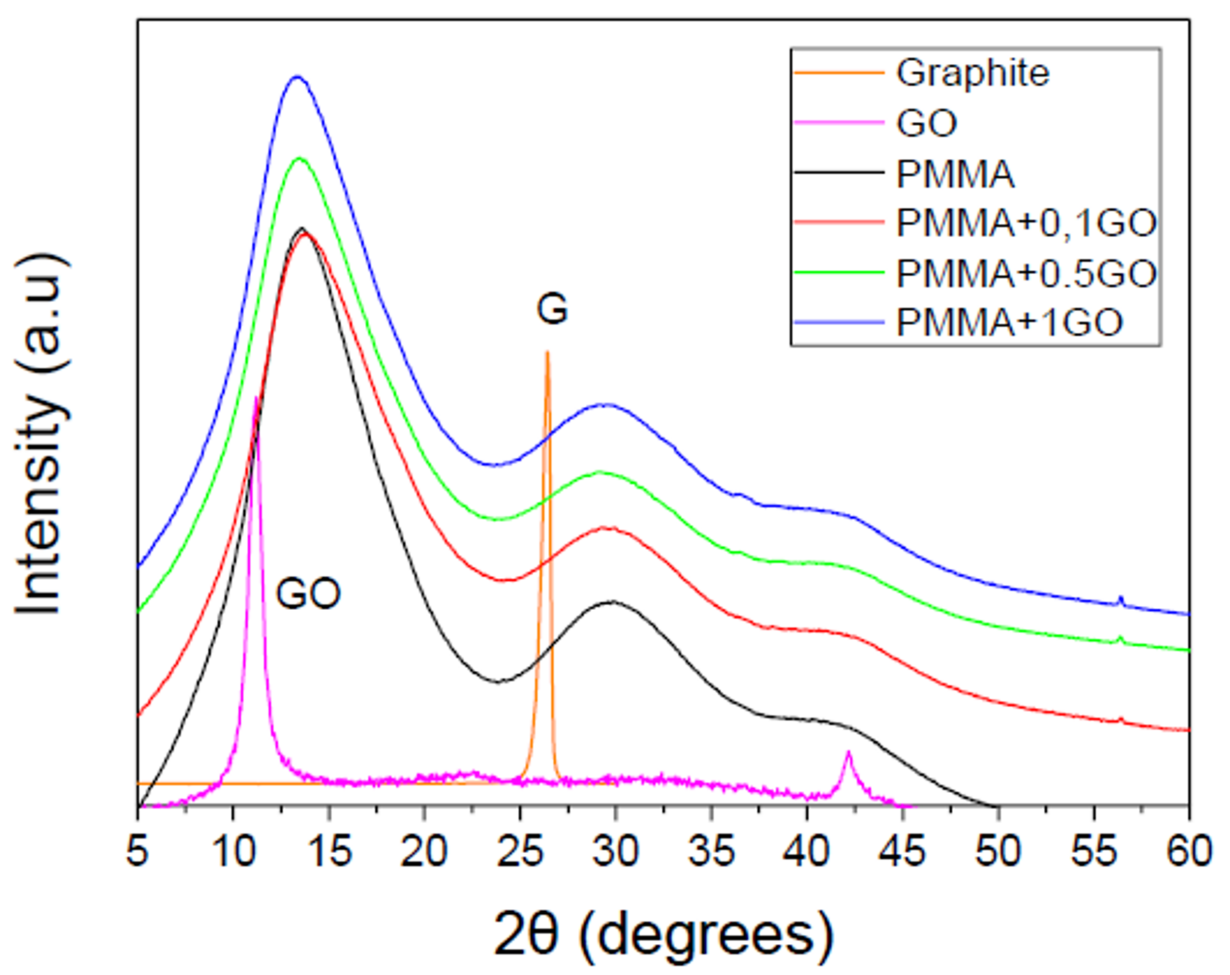
X-ray diffraction patterns of graphite, GO, neat PMMA, and PMMA/GO nanocomposites.

In order to identify the exfoliation of graphite oxide to graphene oxide after polymerization, XRD measurements were carried for graphite, graphite oxide, neat PMMA, and the nanocomposites of PMMA/GO. From the XRD spectra shown in Figure 1, graphite shows a sharp peak at 26.5°. When it is transformed to graphite oxide, this peak is shifted to 11°. Therefore, complete oxidation is verified. Furthermore, the XRD patterns of neat PMMA and PMMA/GO materials were recorded in the angle range of 2θ (5° < 2θ < 60°) and are included in Figure 1. It is seen that in pure PMMA, three very broad peaks appear at 16°, 32°, and 43°, respectively, denoting the amorphous structure of the polymer. When GO was incorporated into the polymer matrix, the same spectrum was recorded, without any obvious peak at 11°. This is an indication that graphite oxide has been exfoliated into graphene oxide during the reaction. TEM measurements could verify these observations.

**Figure 2:**
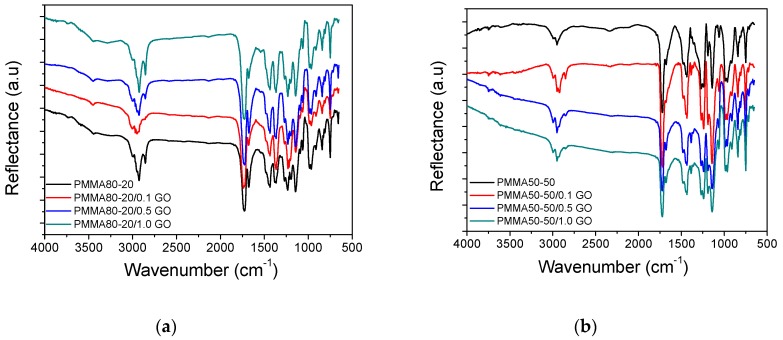
FTIR spectra of neat PMMA and PMMA with 0.1, 0.5, and 1.0% GO obtained under solution polymerization with 80–20 (**a**); 50–50 (**b**) ratio of monomer to solvent.

Possible physicochemical interactions between GO and the PMMA matrix were tested using FTIR-ATR measurements. The FTIR spectra of the material prepared appear in Figure 2. The spectrum of pure PMMA and all the nanocomposites show a sharp peak at 1724 cm^−1^, which corresponds to the carbonyl group, C=O. Two small peaks at 3000/2940 cm^−1^ are attributed to methyl ester C–H stretching vibrations. An additional small peak at 2855 cm^−1^ is due to –CH_3_ stretching vibrations. The peaks at 1436/1482 cm^−1^ correspond to C–H deformations. The peak at 1365 cm^−1^ corresponds to –CH_3_ symmetrical deformation. Finally, the peaks at 1271/1233/1143/985 cm^−1^ are attributed to C–O stretching. Similar reflectance bands have been observed in the FTIR-ATR of pure PMMA in the literature [16]. From Figure 2, the spectra of pure PMMA and all nanocomposites appear similar, indicating that the inclusion of GO in the polymer matrix is rather physical without a strong chemical bond. An analogous observation has been reported in the literature [16].

### 3.2. Polymerization Kinetics

**Figure 3:**
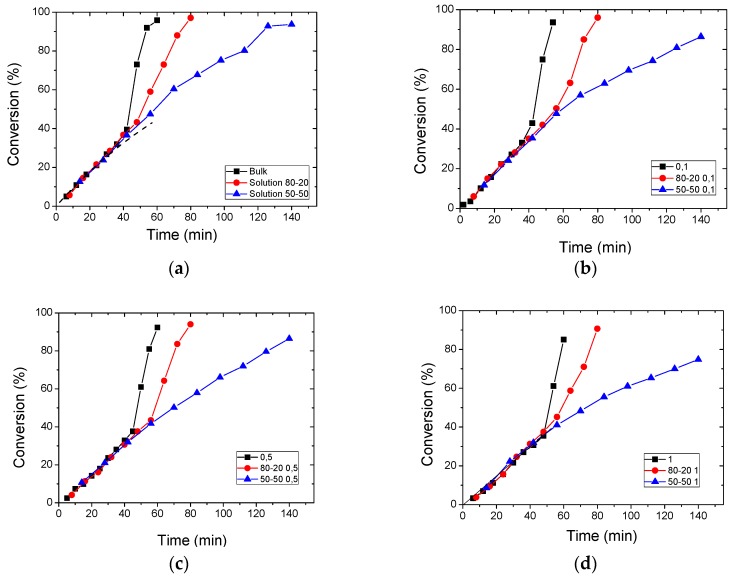
Conversion versus time curves of neat PMMA obtained after bulk and solution polymerization (**a**); PMMA/GO nanocomposites with different relative amounts of GO obtained from in situ bulk polymerization at 80 °C with a 0.03 mol/L initial initiator concentration, 0.1 % (**b**); 0.5% (**c**); 1.0% (**d**).

The effect of carrying out the polymerization in bulk or in solution with two different solvent ratios on the variation of conversion with time is illustrated in Figure 3 for neat PMMA and its nanocomposites with 0.1, 0.5, and 1.0 wt % GO. Conversion time curves approximately follow classical radical polymerization kinetics until near 30% conversion, whereas afterwards, an increase in the reaction rate is observed due to the well-known auto-acceleration or gel-effect. Accordingly, as the reaction proceeds, the movement of macroradicals to find one another to terminate is hindered by the presence of the macromolecular chains. These diffusion-controlled phenomena lead to reduced termination rates of macroradicals, locally increasing their concentration. As a result, this enhanced the reaction rates. During solution polymerization, as the amount of solvent is increased, the auto-acceleration is decreased since macroradicals have more space to move freely and find one another to terminate. Hence, the polymerization rate lowers and more time is needed to complete the reaction. This was observed in both neat PMMA and all nanocomposites (Figure 3). Finally, at conversions higher than 90%, the reaction rate slows down significantly and polymerization almost stops before the full consumption of the monomer. This phenomenon corresponds to the well known glass effect, where the monomer-polymer mixture becomes a “glass”. At this point, diffusion-controlled phenomena also affect the propagation and the initiation reaction. The propagation rate constant and the initiator efficiency decrease significantly and even small molecules (i.e., monomer, primary initiator radicals) cannot easily move in space. Thus, unreacted monomers are trapped without being able to react with the macro-radicals, leaving some residual monomer [17,18,19,20].

**Figure 4:**
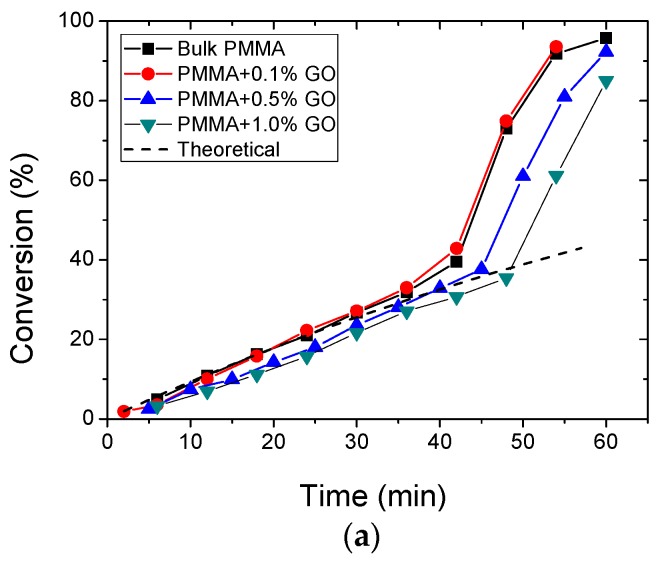

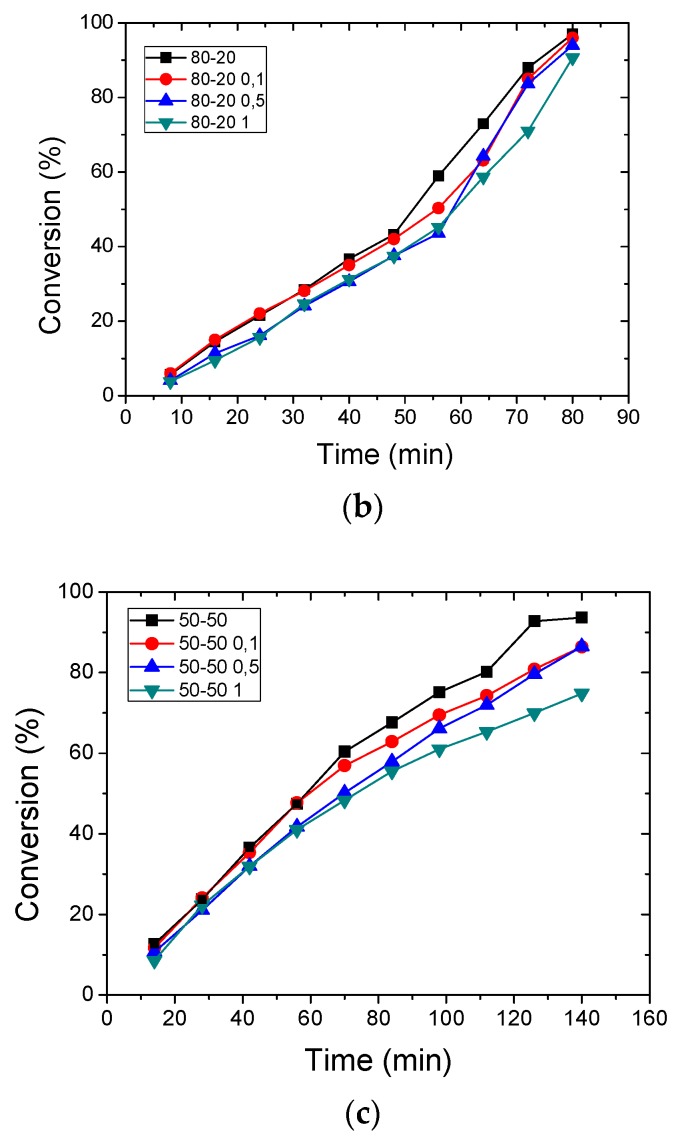
Conversion versus time curves of neat PMMA and PMMA/GO nanocomposites with different relative amounts of GO obtained from in situ polymerization at 80 °C with a 0.03 mol/L initial initiator concentration in bulk (**a**); in solution 80–20 (**b**); 50–50 (**c**).

Furthermore, the effect of adding GO on the PMMA polymerization kinetics is investigated in Figure 4. It is seen that in both bulk and solution polymerization, the behavior of the PMMA with 0.1 wt % GO is very similar to that of neat PMMA, meaning that such a small amount of additive does not influence the reaction kinetics much. However, as the amount of GO added increases, it is clearly observed in all different polymerization conditions that the initial polymerization rate decreases. This has also been observed in the literature in other nano-additives added to PMMA polymerization [14,16].

In order to provide an explanation for the effect of GO on the polymerization kinetics, we used the following Equation (1) for the variation of monomer conversion, *X* with time, *t*. Thus, the polymerization rate, *dX*/*dt*, assuming the steady-state approximation for the total radical concentration (which has been proven to hold at low monomer conversion), is expressed as [18]:

(1) $$\frac{dX}{dt}=\left(k_{p}+k_{trM}\right){\left(\frac{fk_{d}[I]}{k_{t}}\right)}^{1/2}(1-X)$$

where, *k_p_*, *k_trM_*, *k_t_*, and *k_d_* denote the kinetic rate constants of the propagation, chain transfer to monomer, termination, and initiator decomposition reactions, respectively; *f* is the initiator efficiency; and [*I*] is the initiator concentration.

Assuming that the initiator concentration remains almost constant at short reaction times and all kinetic rate constants are independent of conversion, Equation (1) can be integrated to give:

(2) $$X=1-\exp(-k_{eff}t)\;\mathrm{or}\;-\ln(1-X)=k_{eff}t$$

with

(3) $$k_{eff}=\left(k_{p}+k_{trM}\right){\left(\frac{fk_{d}{\left[I\right]}_{0}}{k_{t}}\right)}^{1/2}$$

**Figure 5:**
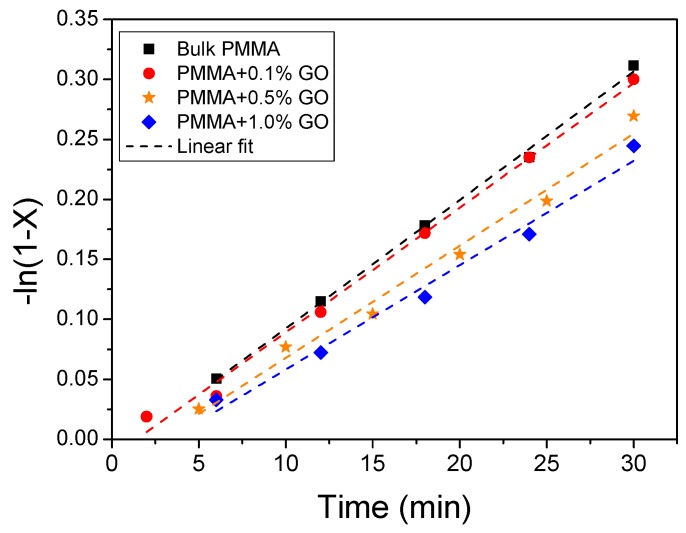
Estimation of the effective rate constant from plots of –ln(1−*X*) versus time according to Equation (2) for the neat PMMA and PMMA/GO nanocomposites with different relative amounts of GO obtained from in situ polymerization at 80 °C with a 0.03 mol/L initial initiator concentration in bulk.

The effective rate constant of PMMA, *k_eff_*, can be evaluated from available literature data on the kinetic rate constant at low conversions. A number of different values have been proposed. In the following, we made use of those reported in a very recent paper by Zoller et al. [21], i.e., *k_p_* = 2.67 × 10^6^*exp(−22,360/RT), *k_t_* = 1.984 × 10^8^*exp(−5,890/RT) L/mol/s, *k_d_* = 5 × 10^16^*exp(−143,000/RT)·s^−1^, *k_trM_* = 5 × 10^−5^*k_p_, and *f* = 0.5. The values of the above kinetic rate constants at 80 °C, where the experiments of this study were carried out, are: *k_p_* = 1314 L/mol/s, *k_t_* = 2.668 × 10^7^ L/mol/s, *k_d_* = 3.5 × 10^−5^ s^−1^, *k_trM_* = 0.066 L/mol/s. Using these values and [*I*]_0_ = 0.03 mol/L, the theoretical value of the effective rate constant becomes, *k_eff_* = 1.84 × 10^−4^ s^−1^ or 1.106 × 10^−2^ min^−1^. Then, by means of Equation (2), the variation of conversion with time at low conversions can be estimated and is included as a continuous line in Figure 3a. It is seen that the theoretical line simulates the experimental data very well at low conversions. The next step was to identify which kinetic parameter is affected by the addition of GO. From Equation (1), it is unlikely that *k_p_*, *k_trM_*, *k_t_*, or *k_d_* would change with the existence of the GO. Then, it seems that the initiator efficiency, f, is affected and particularly decreases with an increasing nano-additive content. Furthermore, the effective rate constant, *k_eff_*, can be estimated using Equation (2) from the slope of the curve obtained after plotting –ln(1−*X*) vs. *t*. Such curves for the bulk polymerization of PMMA and its nanocomposites with GO appear in Figure 5. The corresponding curves for the solution polymerizations were similar to those shown in this figure, since as it can be seen in Figure 3, the data at the same amount of GO and at conversions less than 30% (as we used here) are very similar. The estimated values of *k_eff_*, together with their standard error and correlation coefficient, appear in Table 1. As it can be seen, very clear straight lines were obtained for all curves. From the values of *k_eff_* and using Equation (3) with the aforementioned parameter values, the initiator efficiencies, *f*, were estimated and are included in Table 1. It can be seen that the value of f for neat PMMA, i.e., 0.47, is very close to the theoretical (literature) value of 0.5. Moreover, a clear decrease of the initiator efficiency with the increasing amount of GO was observed. This is a clear indication that graphene oxide acts as a scavenger of primary initiator radicals at the early stages of polymerization. This is in accordance with the results presented in our previous work [15,16] for in situ MMA homopolymerization in the presence of nano-additives such as organomodified montmorillonites.

**Scheme 1:**
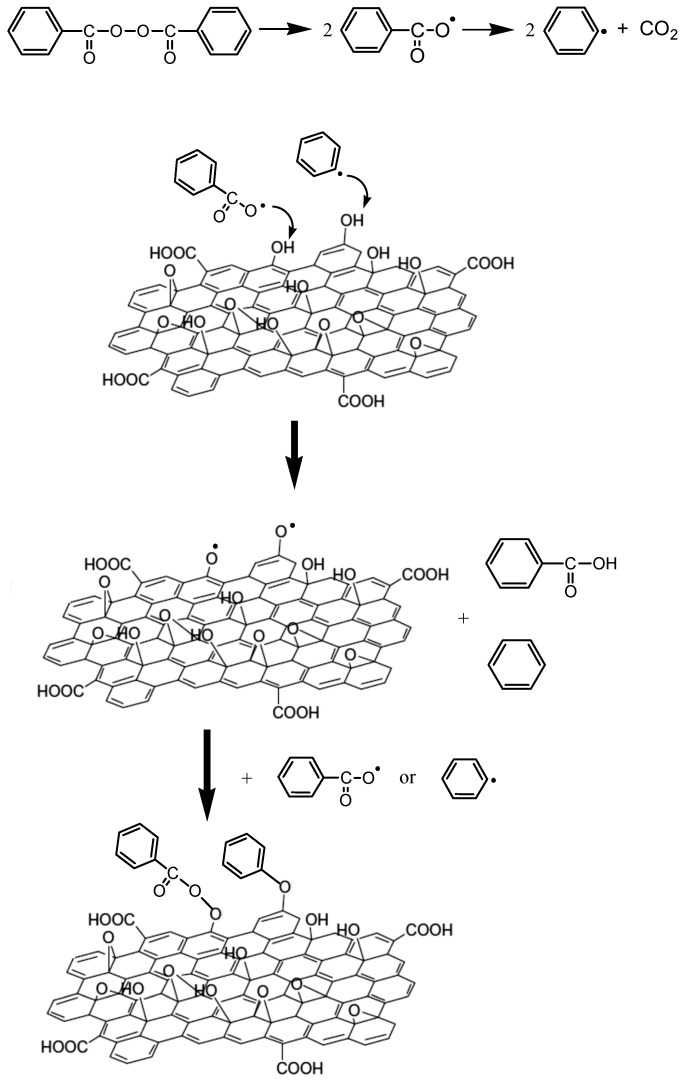
Schematic illustration of the reaction of primary initiator radicals with GO resulting in their deactivation and reduction of the initiator efficiency.

In order to provide an explanation for the reduced initiator efficiency with the nano-additive content, the decomposition of the initiator used, i.e., BPO, is considered in Scheme 1. Accordingly, two benzoyloxy radicals are initially produced, which can further decompose to phenyl radicals and carbon dioxide. Both primary radicals formed from the decomposition of the initiator may react with the phenolic hydroxyls on the GO surface by abstracting a hydrogen atom. The phenoxy radicals may then scavenge a further radical (Scheme 1). Thus, one or two primary radicals may be terminated for every mole of phenolic OH. As a result, the effective number of primary radicals formed from the fragmentation of the initiator, which can find a monomer molecule and start polymerization, is decreased.

**Figure 6:**
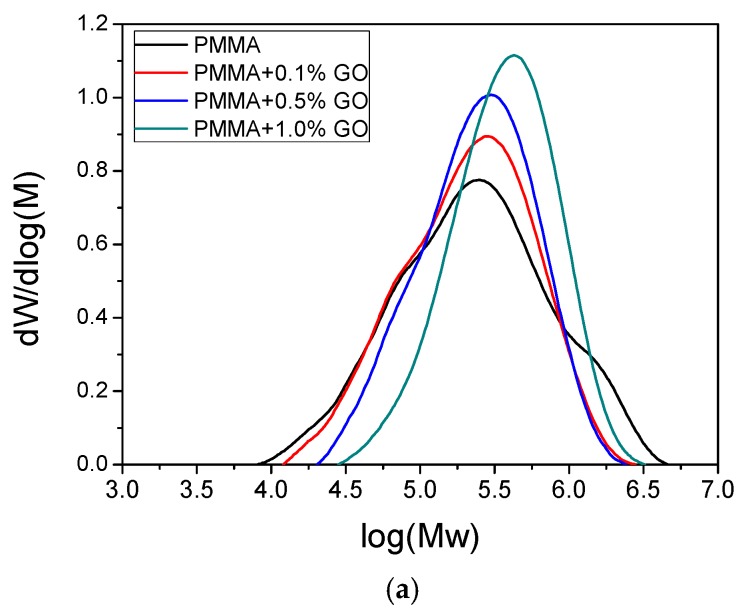

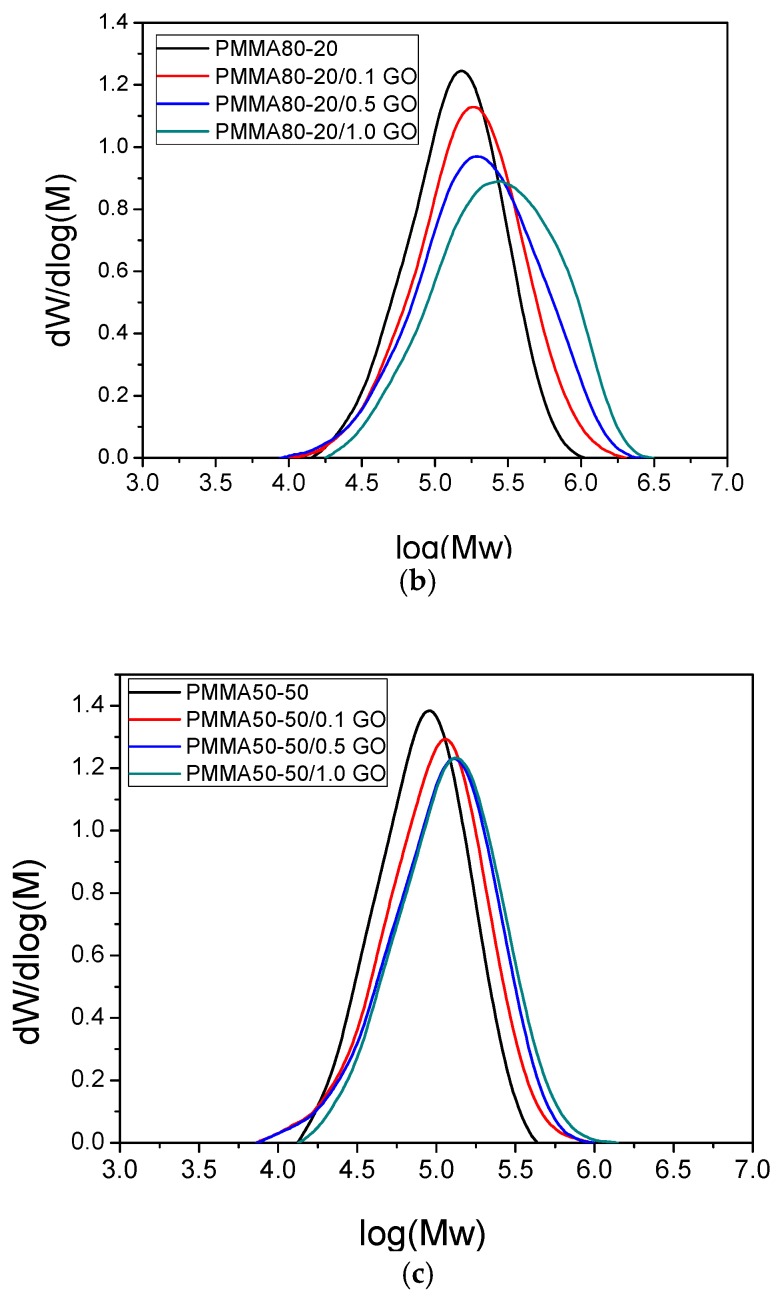
Full Molecular weight distribution of neat PMMA and PMMA/GO nanocomposites obtained in bulk (**a**) and solution with a 80–20 (**b**) and 50–50 (**c**) ratio of monomer to solvent.

Furthermore, the full molecular weight distribution of neat PMMA and PMMA/GO nanocomposites obtained in bulk and solution with a 80–20 and 50–50 ratio of monomer to solvent, measured via GPC, appears in Figure 6. The average molecular weights of PMMA and all nano-hybrids prepared are illustrated in Table 2. It was observed that the average molecular weight, *M*_n_, of the material formed increases when increased amounts of GO are added. Moreover, from Figure 6, the formation of polymers with a narrower MWD distribution was revealed when GO was added. To explain these measurements we returned again to classical free radical polymerization kinetics. Accordingly, the average molecular weight of a polymer is given by its average degree of polymerization, which in turn is calculated from the average kinetic chain length, *ν*, knowing the mode of termination by combination or disproportionation. ν can be calculated from the following equation:

(4) $$\frac{1}{\nu }=\frac{k_{t}[P^{•}]}{k_{p}[M]}+\frac{k_{trM}[M]}{k_{p}[M]}=\frac{{\left(fk_{d}[I]k_{t}\right)}^{1/2}}{k_{p}[M]}+C_{M}$$

with *C_M_* = *k_trM_*/*k_p_*.

According to Equation (4), the average kinetic chain length, *ν*, is inversely proportional to the initiator efficiency, *f*. Therefore, when *f* decreases, *ν*, and as a result, the average molecular weight of the polymer, increases. The physical meaning of this is that macro-radicals with a higher chain length are produced when the number of primary initiator radicals is decreased. Moreover, it was found that higher amounts of GO result in a lower final conversion, which in turn stops the polymer from increasing its high molecular weight tail to higher values, resulting in the reduced polydispersity of the MWD.

**Figure 7:**
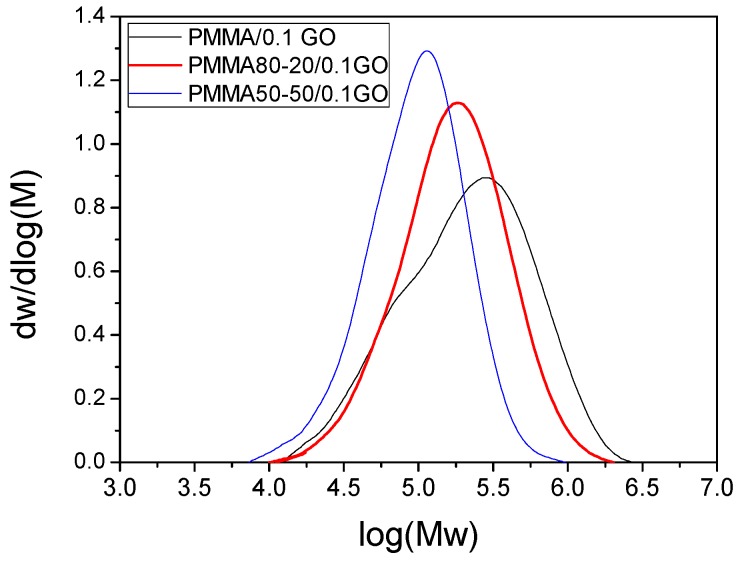
Full Molecular weight distribution of neat PMMA obtained after bulk and solution polymerization.

Moreover, carrying out the polymerization in the presence of solvent results in polymers with significantly reduced average molecular weights (Table 2). As it is seen in Figure 7, the whole MWD shifts to lower values. This is a direct result of Equation (4), where it is clear that as the monomer concentration is decreased (a higher monomer concentration is achieved in bulk polymerization), the kinetic chain length is also decreased. Thus, higher amounts of solvent results in a reduced average molecular weight of the polymer. Another reason is that, as it was observed in Figure 3, the presence of the solvent suppresses the gel-effect. In the presence of strong autoacceleration (effect of diffusion-controlled phenomena), the average molecular weights of the polymer increase with monomer conversion. Since adding a solvent reduces the effect of diffusion controlled phenomena, it also results in a reduced increase of *M*_n_ and *M*_w_ with monomer conversion, and as a result, in lower final values.

### 3.3. Thermal Properties of the Nanocomposites

**Figure 8:**
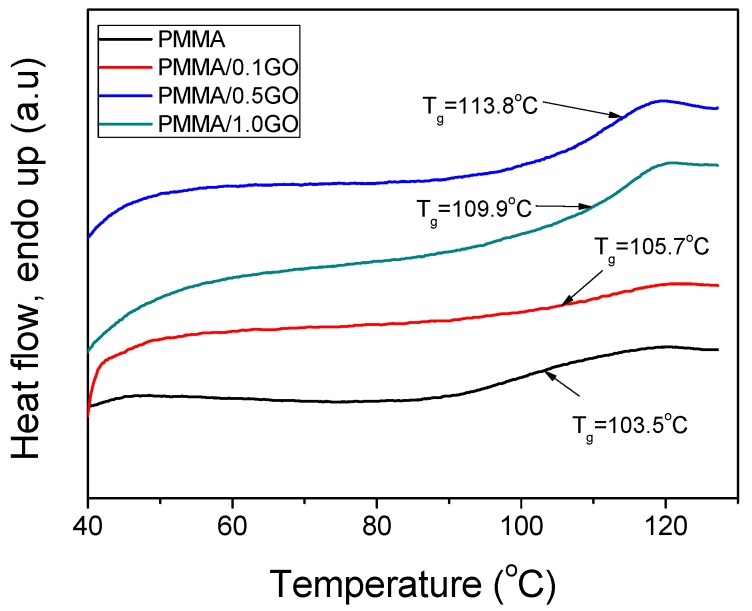
DSC traces of PMMA and all PMMA/GO nanocomposites, to estimate their glass transition temperatures.

DSC was used to measure the glass transition temperature, *T*_g_, of neat PMMA and the nano-hybrids, according to the procedure described in the experimental section and the half C_p_ extrapolation method [9]. Indicative results of DSC traces obtained from the bulk polymerization experiments are shown in Figure 8. The *T*_g_ values estimated are given in Table 2. For pristine PMMA, a value near 103.5 °C was recorded, close to that reported in the literature (near 100 °C [9]). When the amount of GO added was increased to 0.1, 0.5, and 1.0 wt %, the *T*_g_ of the polymer was increased to 105.7, 109.9, and 113.8 °C, respectively. The same tendency was observed in the solution experiments with the *T*_g_ increasing from 86.6 to 92.9 °C and from 82.2 to 90.9 °C for the 80–20 and 50–50 ratios, respectively. The interaction of polymer chains and nano-particles at the surface can alter the chain kinetics by either decreasing or increasing the glass transition temperature of the polymer [12]. The enhancement in the *T*_g_ of the nanocomposites could be attributed to the restriction in chain mobility due to the confinement effect of 2D-layered graphene incorporated into the matrix and the strong nanofiller—polymer interactions.

**Figure 9:**
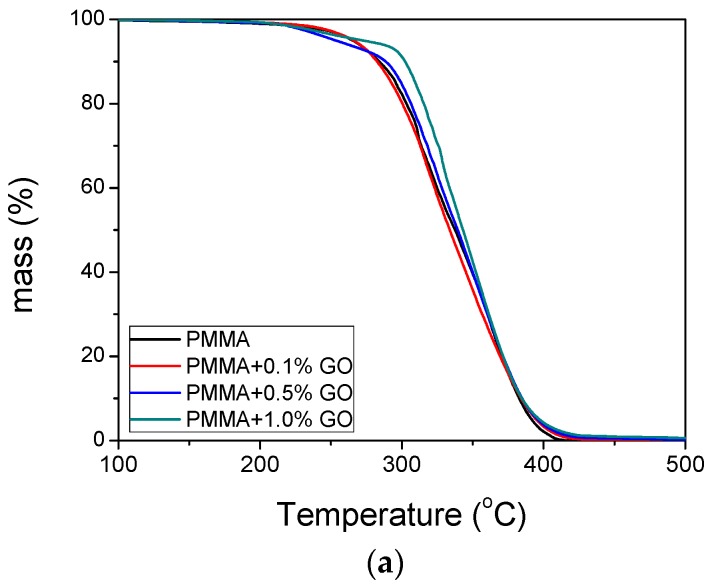

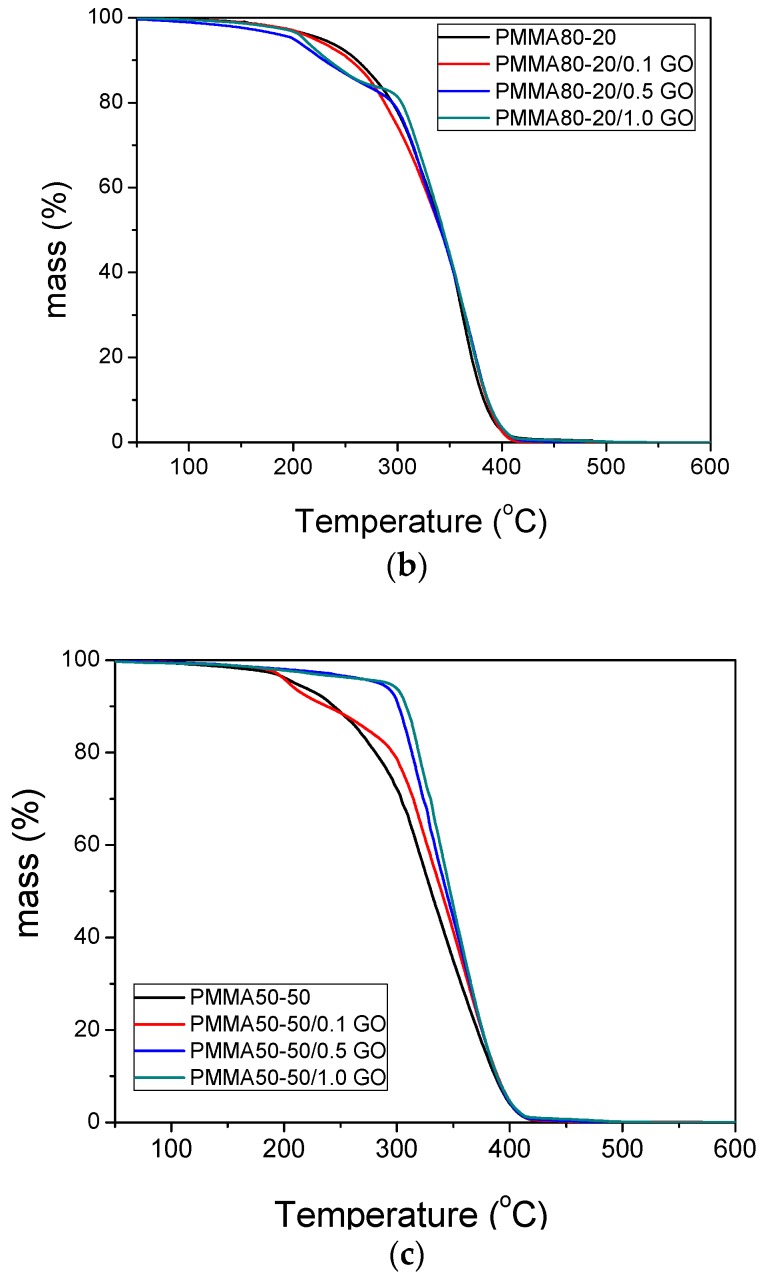
Mass loss of neat PMMA and PMMA/GO nanocomposites with various amounts of added GO obtained in bulk (**a**); solution with 80–20 (**b**); 50–50 (**c**) ratio of monomer to solvent.

In addition, the thermal stability of pure PMMA and PMMA/GO nanocomposites was examined by thermogravimetric analysis in an inert (nitrogen) atmosphere. Results on the variation of mass loss with an increasing temperature of neat polymer and the nano-hybrids with various amounts of added GO obtained after bulk or solution polymerization are illustrated in Figure 9a–c. The thermal degradation of radically prepared PMMA has been a subject of numerous studies and usually involves multiple steps assigned to: the scission of unsaturated terminal groups, presence of weak head-to-head linkages, and random scission of the carbon-carbon main chain. It is generally considered that most PMMA thermally degrades through depolymerisation. In addition, it has been shown that GO exhibits a significant mass loss (almost 22%) at 210–250 °C, which is due to the pyrolysis of labile oxygen-containing functional groups, such as –COOH, –OH, etc. The incorporation of GO in the polymer matrix results in an enhanced thermal stability of the resulting nanocomposite compared to neat polymer, especially when higher amounts of GO are incorporated [22]. Therefore, the mass loss observed in Figure 9 in the region of 210–250 °C is attributed to the elimination of labile functional groups from the surface of GO. Moreover, the incorporation of 0.1 wt % GO does not seem to show any significant effect. However, the addition of higher GO amounts seems to increase the thermal stability of the polymer formed, which was more pronounced in the PMMA nanocomposites obtained after solution 50–50 polymerization and is shown in Figure 9c. Finally, thermal degradation seems to start earlier in the polymers and nanocomposites formed after solution polymerization compared to corresponding from bulk. This is mainly attributed to the lower average molecular weight of the polymers produced via solution polymerization compared to those from bulk.

## 4. Conclusions

Nanocomposite materials of poly(methyl methacrylate) with GO were produced using the in situ radical polymerization technique carried out in bulk and solution at different solvent/monomer ratios, from graphite oxide, the MMA monomer, and benzoyl peroxide, the initiator. FTIR data showed that the inclusion of GO in the polymer matrix was rather physical, without a strong chemical bond. XRD data showed that graphite oxide had been transformed to graphene oxide during polymerization. From the study of the reaction kinetics, it was found that the initiator efficiency is reduced by the presence of the GO, resulting in a reduction of the reaction rate and a slight increase in the average molecular weight of the polymer formed. A polymer with a lower average molecular weight was produced when polymerization was carried out in solution, again increasing with the amount of GO added. The polydispersity of the MWD was found to decrease with the amount of solvent added. Moreover, the glass transition temperature of the polymer was increased with the amount of GO added, whereas it was decreased during solution polymerization. Finally, from thermogravimetric analysis, it was verified that the presence of GO results in materials with a slightly higher thermal stability.

## Figures and Tables

**Table 1:** Effective rate constant, *k_eff_*, estimated from the slope of the curves presented in Figure 5 of neat PMMA and its nanocomposites with various amounts of GO obtained in bulk polymerization and corresponding initiator efficiencies estimated from Equation (3).

Sample	*k_eff_* (min^−1^)	Standard Error	*R*^2^	*f*
PMMA	0.0107	2.68 × 10^−4^	0.9975	0.47
PMMA/0.1GO	0.01038	3.75 × 10^−4^	0.9935	0.44
PMMA/0.5GO	0.00935	5.65 × 10^−4^	0.9820	0.36
PMMA/1.0GO	0.0087	6.24 × 10^−4^	0.9797	0.31

**Table 2:** Number-average, weight-average, and z-average molecular weights (${\bar{M}}_{n}$, ${\bar{M}}_{w}$, ${\bar{M}}_{z}$), polydispersity of the MWD (PD) and glass transition temperature (*T*_g_) of neat PMMA and its nanocomposites with various amounts of GO obtained in bulk or solution polymerization with a 80–20 and 50–50 ratio of monomer to solvent.

Sample	${\bar{M}}_{n}$	${\bar{M}}_{w}$	${\bar{M}}_{z}$	PD	*T*_g_
PMMA	114,570	427,250	1,121,690	3.73	103.5
PMMA/0.1GO	124,024	321,516	626,860	2.59	105.7
PMMA/0.5GO	160,872	339,196	592,744	2.11	109.9
PMMA/1.0GO	255,553	480,359	781,407	1.88	113.8
PMMA80–20	102,673	173,944	267,436	1.69	86.6
PMMA80–20/0.1GO	119,534	233,037	409,315	1.95	88.0
PMMA80–20/0.5GO	144,446	333,565	634,007	2.31	91.1
PMMA80–20/1.0GO	168,209	390,492	723,507	2.32	92.9
PMMA50–50	65,699	98,390	138,636	1.50	82.2
PMMA50–50/0.1GO	71,766	123,872	193,358	1.73	85.8
PMMA50–50/0.5GO	77,709	140,321	218,256	1.81	88.0
PMMA50–50/1.0GO	91,579	156,504	250,996	1.71	90.9
